# Supplementary material for: A Meta-Analysis of Wearable Contact Lenses for Medical Applications: Role of Electrospun Fiber for Drug Delivery
Source: Polymers (Basel). 2022 Jan 3;14(1):185. doi: 10.3390/polym14010185 (PMC8747307; doi:10.3390/polym14010185)
Supplement: Supplementary file 1 [file polymers-14-00185-s001.zip › polymers-1518216-supplementary.pdf]

Supplementary Section

A Meta-Analysis of Wearable Contact Lenses for Medical Applications: Role of Electrospun Fiber for Drug Delivery

Hamed Hosseinian [1], Samira Hosseini [1,2,*], Sergio O. Martinez-Chapa [1] and Mazhar Sher [3,*]

1 School of Engineering and Sciences, Tecnologico de Monterrey, Ave. Eugenio Garza Sada 2501, Monterrey 64849, Mexico; A00831053@itesm.mx (H.H.); smart@tec.mx (S.O.M.-C.)
2 Writing Lab, Institute for the Future of Education, Tecnologico de Monterrey, Monterrey 64849, Mexico
3 Department of Mechanical Engineering and Applied Mechanics, School of Engineering and Applied Science, University of Pennsylvania, Philadelphia, PA 19104, USA
* Correspondence: samira.hosseini@tec.mx (S.H.); mazhars@seas.UPenn.edu (M.S.); Tel.: +52-8123267576 (S.H.)

Supplementary Annex S1. The table of query for finding the relevant articles within Scopus database.

Area	Query String	The number of articles in Scopus database
IOP measurement	TITLE-ABS-KEY (contact AND lens) OR TITLE-ABS-KEY (iop) AND TITLE-ABS-KEY intraocular AND pressure) OR TITLE-ABS-KEY (OPA) AND (LIMIT-TO (PUBSTAGE,"final")) AND (LIMIT-TO SUBJAREA,"MEDI") OR LIMIT-TO (SUBJAREA,"CENG") OR LIMIT-TO (SUBJAREA,"BIOC") OR LIMIT-TO SUBJAREA,"PHAR")) AND (LIMIT-TO (PUBYEAR,2022) OR LIMIT-TO (PUBYEAR,2021) OR LIMIT-TO (PUBYEAR,2020) OR LIMIT-TO (PUBYEAR,2019) OR LIMIT-TO (PUBYEAR,2018) OR LIMIT-TO (PUBYEAR,2017) OR LIMIT-TO (PUBYEAR,2016) OR LIMIT-TO (PUBYEAR,2015) OR LIMIT-TO (PUBYEAR,2014) OR LIMIT-TO (PUBYEAR,2013) OR LIMIT-TO (PUBYEAR,2012) OR LIMIT-TO (PUBYEAR,2011) OR LIMIT-TO (PUBYEAR,2010) OR LIMIT-TO (PUBYEAR,2009) OR LIMIT-TO (PUBYEAR,2008) OR LIMIT-TO (PUBYEAR,2007) OR LIMIT-TO (PUBYEAR,2006) OR LIMIT-TO (PUBYEAR,2005) OR LIMIT-TO (PUBYEAR,2004) OR LIMIT-TO (PUBYEAR,2003) OR LIMIT-TO (PUBYEAR,2002) OR LIMIT-TO (PUBYEAR,2001) OR LIMIT-TO (PUBYEAR,2000))	18879
Glucose measurement and monitoring	TITLE-ABS-KEY (contact lens) AND TITLE-ABS-KEY (glucose) OR TITLE-ABS-KEY (diabetes) AND (LIMIT-TO (PUBSTAGE,"final")) AND (LIMIT-TO (SUBJAREA,"MEDI") OR LIMIT-TO (SUBJAREA,"CENG") OR LIMIT-TO (SUBJAREA,"BIOC") OR LIMIT-TO (SUBJAREA,"PHAR")) AND (LIMIT-TO (PUBYEAR,2022) OR LIMIT-TO (PUBYEAR,2021) OR LIMIT-TO (PUBYEAR,2020) OR LIMIT-TO (PUBYEAR,2019) OR LIMIT-TO (PUBYEAR,2018) OR LIMIT-TO (PUBYEAR,2017) OR LIMIT-TO (PUBYEAR,2016) OR LIMIT-TO (PUBYEAR,2015) OR LIMIT-TO (PUBYEAR,2014) OR LIMIT-TO (PUBYEAR,2013) OR LIMIT-TO (PUBYEAR,2012) OR LIMIT-TO (PUBYEAR,2011) OR LIMIT-TO (PUBYEAR,2010) OR LIMIT-TO (PUBYEAR,2009) OR LIMIT-TO (PUBYEAR,2008) OR LIMIT-TO (PUBYEAR,2007) OR LIMIT-TO (PUBYEAR,2006) OR LIMIT-TO (PUBYEAR,2005) OR LIMIT-TO (PUBYEAR,2004) OR LIMIT-TO (PUBYEAR,2003) OR LIMIT-TO (PUBYEAR,2002) OR LIMIT-TO (PUBYEAR,2001) OR LIMIT-TO (PUBYEAR,2000))	403

Colorblindness	TITLE-ABS-KEY (colorblindness) OR TITLE-ABS-KEY (CVD) OR TITLE-ABS-KEY (color vision deficiency) AND TITLE-ABS-KEY (contact lens) AND (LIMIT-TO (PUBSTAGE,"final")) AND (LIMIT-TO (DOCTYPE,"ar")) AND (LIMIT-TO (SUBJAREA,"MEDI") OR LIMIT-TO (SUBJAREA,"CENG") OR LIMIT-TO (SUBJAREA,"BIOC") OR LIMIT-TO (SUBJAREA,"PHAR")) AND (LIMIT-TO (PUBYEAR,2022) OR LIMIT-TO (PUBYEAR,2021) OR LIMIT-TO (PUBYEAR,2020) OR LIMIT-TO (PUBYEAR,2019) OR LIMIT-TO (PUBYEAR,2018) OR LIMIT-TO (PUBYEAR,2017) OR LIMIT-TO (PUBYEAR,2016) OR LIMIT-TO (PUBYEAR,2015) OR LIMIT-TO (PUBYEAR,2014) OR LIMIT-TO (PUBYEAR,2013) OR LIMIT-TO (PUBYEAR,2012) OR LIMIT-TO (PUBYEAR,2011) OR LIMIT-TO (PUBYEAR,2010) OR LIMIT-TO (PUBYEAR,2009) OR LIMIT-TO (PUBYEAR,2008) OR LIMIT-TO (PUBYEAR,2007) OR LIMIT-TO (PUBYEAR,2006) OR LIMIT-TO (PUBYEAR,2005) OR LIMIT-TO (PUBYEAR,2004) OR LIMIT-TO (PUBYEAR,2003) OR LIMIT-TO (PUBYEAR,2002) OR LIMIT-TO (PUBYEAR,2001) OR LIMIT-TO (PUBYEAR,2000))	11
Drug delivery	TITLE-ABS-KEY (drug delivery) AND TITLE-ABS-KEY (contact lens) AND (LIMIT-TO (PUBSTAGE,"final")) AND (LIMIT-TO (SUBJAREA,"MEDI") OR LIMIT-TO (SUBJAREA,"CENG") OR LIMIT-TO (SUBJAREA,"BIOC") OR LIMIT-TO (SUBJAREA,"PHAR")) AND (LIMIT-TO (PUBYEAR,2022) OR LIMIT-TO (PUBYEAR,2021) OR LIMIT-TO (PUBYEAR,2020) OR LIMIT-TO (PUBYEAR,2019) OR LIMIT-TO (PUBYEAR,2018) OR LIMIT-TO (PUBYEAR,2017) OR LIMIT-TO (PUBYEAR,2016) OR LIMIT-TO (PUBYEAR,2015) OR LIMIT-TO (PUBYEAR,2014) OR LIMIT-TO (PUBYEAR,2013) OR LIMIT-TO (PUBYEAR,2012) OR LIMIT-TO (PUBYEAR,2011) OR LIMIT-TO (PUBYEAR,2010) OR LIMIT-TO (PUBYEAR,2009) OR LIMIT-TO (PUBYEAR,2008) OR LIMIT-TO (PUBYEAR,2007) OR LIMIT-TO (PUBYEAR,2006) OR LIMIT-TO (PUBYEAR,2005) OR LIMIT-TO (PUBYEAR,2004) OR LIMIT-TO (PUBYEAR,2003) OR LIMIT-TO (PUBYEAR,2002) OR LIMIT-TO (PUBYEAR,2001) OR LIMIT-TO (PUBYEAR,2000))	588
Electrospun fiber	TITLE-ABS-KEY (electrospun fiber) OR TITLE-ABS-KEY (electrospinning) OR TITLE-ABS-KEY (fiber) AND TITLE-ABS-KEY (contact lens) AND (LIMIT-TO (PUBSTAGE,"final")) AND (LIMIT-TO (DOCTYPE,"ar")) AND (LIMIT-TO (SUBJAREA,"MEDI") OR LIMIT-TO (SUBJAREA,"CENG") OR LIMIT-TO (SUBJAREA,"BIOC") OR LIMIT-TO (SUBJAREA,"PHAR")) AND (LIMIT-TO (PUBYEAR,2022) OR LIMIT-TO (PUBYEAR,2021) OR LIMIT-TO (PUBYEAR,2020) OR LIMIT-TO (PUBYEAR,2019) OR LIMIT-TO (PUBYEAR,2018) OR LIMIT-TO (PUBYEAR,2017) OR LIMIT-TO (PUBYEAR,2016) OR LIMIT-TO (PUBYEAR,2015) OR LIMIT-TO (PUBYEAR,2014) OR LIMIT-TO (PUBYEAR,2013) OR LIMIT-TO (PUBYEAR,2012) OR LIMIT-TO (PUBYEAR,2011) OR LIMIT-TO (PUBYEAR,2010) OR LIMIT-TO (PUBYEAR,2009) OR LIMIT-TO (PUBYEAR,2008) OR LIMIT-TO (PUBYEAR,2007) OR LIMIT-TO (PUBYEAR,2006) OR LIMIT-TO (PUBYEAR,2005) OR LIMIT-TO (PUBYEAR,2004) OR LIMIT-TO (PUBYEAR,2003) OR LIMIT-TO (PUBYEAR,2002) OR LIMIT-TO (PUBYEAR,2001) OR LIMIT-TO (PUBYEAR,2000))	202

Total number of articles	20083

Table S1. Contact lenses for IOP measurement and monitoring. The table summarizes all the existing platforms including their key parts, the process of fabrication, the operation mechanism, the working principle and development stage, as well as the advantages and the disadvantages.

Main components	Fabrication strategy	Mechanism of operation	Application	Working principle	Development stage	Advantages	Disadvantages or limitations	Refs.
**PET-based contact lens**								
Wheatstone circuit<br>PET<br>Two counterpart active strain gauges<br>Two counterpart passive strain gauges	Photolithography was applied by exposure of the samples to mercury radiation. Oxygen plasma was used to eliminate the residual photoresist. Subsequently, a Ti and a Pt layer were deposited, and the LIFT-OFF technique was applied on PET film. The contact lens was made through a thermal mold, and the lens was encapsulated in a biocompatible PDMS layer.	A piezo-resistive strain gauge detects the deformation, which results in decrease/increase of resistance. Wheatstone bridge measures the weak signal variation through and represents a linear relationship between the output voltage and IOP variation. To simulate the eye pressure, a syringe pump utilized to extract water from silicone eye for pressure decrease and to inject water for pressure increase.	IOP monitoring	Piezo-resist mechanism	Tested on silicone eye model.	It is non-invasive and for various speeds of IOP pressure fluctuations, it demonstrates an admirable dynamic response.	The sensor's complex structure may impact the lens's rigidity, hence inducing sensitivity towards the corneal layer.	[1]
**IOP-glucose monitoring contact lens**								
Graphene<br>Hybrid metal nanowires<br>Resistance circuit<br>Inductance circuit<br>Capacitance circuit	The plastic substrate such as PETE and PDMS were bent on cylindrical supports with various radii of curvature. The developed lens included graphene, CNTs, mNWs, metal mesh structures, and the AgNW hybrid structure as stretchable, transparent electrodes. Graphene was transferred onto the AgNW electrodes and patterned by photolithography and RIE was used for the hybrid electrodes and	Among the three elements in the demonstrated RLC circuit, resistance responds to molecular binding, while inductance and capacitance vary with structural changes of the device, thus enabling the detection of IOP.	Glucose and IOP monitoring	By an electrical resistance-inductance-capacitance resonant circuit	For IOP measurement tested *in-vitro* on a bovine eyeball and for Glucose monitoring *in-vivo* tested on a male New Zealand white rabbit.	Elective binding of target analytes to graphene tunable to a range of biomarkers such as glucose was made possible.	Precise diagnosis of glucose may require further development of the sensor.	[2]

	channel.						
**MATEO ANR (named after a deceased student colleague)**							
Passive sensor Spectacle frame with em-bedded electron-ics Recording device	The process of fabrication for pressure sensors was conducted via MEMS technology. A microelectronic wafer fabrication method provided to fit with the optimum electrical requirements and mechanical considerations to fabricate a contact lens integrated with a passive sensor and resonant circuitry.	The maximum sensitivity of contact lens comes from the structure of sensor that propose covering small cornea fluctuations like even a few tenths of microns. To reach such sensitivity, the combination of antenna on a small transparent and soft polymer lens is necessary.	IOP monitori ng	Under magnetic coupling conditio ns, the eye's mechani cal modifica tions coming from IOP changes transferr ed to the sensor providin g a detectabl e electrical paramet er change.	Tested on isolated pig eyes.	A thinner contact lens with less complicated IOP passive sensor provides a short-time diagnostic mode like every 10 or 5 min for clinicians.	In the pig eye model, the authors increased the chance of error by injecting the solution in the anterior chamber instead of the posterior chamber, as it produced smaller IOP variations.	[3]
**Implantable microfluidic contact lens**							
Airtight microflu-idic chan-nel Gas res-ervoir Optical adaptor	The 8-mm diameter IOP sensor chip was developed from PDMS via standard soft lithography methods. The parylene–C–coated chip and glass weredried by air and left at room temperature for a whole night to make a uniform and robust bond, afterwards.	Intraocular fluid fills the sensor channel till the pressure between IOP and the air inside the channels is equilibrated. A camera or a smartphone provided with an optical and illumination adaptor captures the gas-liquid interface. Image processing is capable for detection of gas-fluid interface, also calculation of IOP pressure and recording for follow-up, which can be transferred to the patient's eye caregivers in case of	IOP monitori ng	A smartph one with an optical readout and analysis segment s recorded and analyzed the signal using embedd ed software .	Successful ly implanted into enucleate d porcine eyes.	A detection limit of 1 mm Hg, excellent reproducibil ity, and noticeable sensitivity was reported for the device.	The problem of optically measuring IOP pressure over a hazy cornea was reported as one of the shortcomings of this device. Additionally, measurement errors can comes from gas leakage through the sensor walls.	[4]

	necessity.							
**Silicone contact lens with an embedded MEMS strain gage**								
Active and passive gages Connecting wires ZIF connector	In order to have the insulation, protection, and creation of a flexible carrier material, the gages were utilized between two layers of polyimide. The device was surface micromachined on a carrier substrate.	The Wheatstone bridge was stimulated via a DC current, which gave a signal Vm (voltage) proportional to its strain. The contact lens slides on the cornea surface bends and follows corneal deformations.	IOP monitoring	The readout was performed by two active (double sensitivity) and two passive gages (thermal compensation).	Tested on an eye-simulating device.	No anesthesia was required, and the device was sensitive enough to measure small variations in cornea curvature.	ZIF connector is unpleasant to be carried by the patient. Moreover, the hydrophobicity of silicone made it challenging to maintain contact with the simulated eye.	[5]
**Capacitive wearable contact lens sensor**								
Capacitor electrodes Inductive coil Reference layer Sensing layer	An inductor-capacitor sensor, which is a type of curvature-sensitive, was developed via μTM. A thin 10 μm copper foil was etched to from the capacitor electrodes and inductive coil. The inductive coil and capacitor electrodes were covered and separately cast inside the silicone layer through mold cast and treated in an oven at 40 ◦C. Finally, adhesive silicone rubber was used on their edges to shape the contact lens sensor.	To develop an LC resonant circuit, the curvature-sensing capacitor was integrated with an inductive coil plus fixed inductance. The resonance frequency and capacitance of the resonant circuit is changed by the fluctuations in the curvature of the soft cornea and contact lens. A reader in a glass frame worn by the user could measure the resonance frequency wirelessly.	IOP monitoring	The resonance frequency of a curvature-sensitive inductor-capacitor was detected.	Tested *ex-vivo* on porcine eyes.	The new strategy uses capacitive sensing, which is more appropriate for low force applications, in spite of depending on strain sensing elements to transform the force into a characteristic signal.	The user must always wear an eyeglass frame to measure the IOP wirelessly.	[6]
**Sensing contact lens**								
Compensation resistive gages Sensing resistive gages Micro-flex connection cable ZIF con-	Cast-molding method was used to fabricate a soft contact lens with an embedded micro-strain gauge. A thin metallic film patterned by surface micromachining on a polyimide microflex substrate utilized to develop the gauges.	Soft contact lens finds a stable balanced position after each blink, bends, and follows corneal deformations. The gauges that are responsible for IOP measurement were fabricated in a	IOP monitoring	Wheatstone bridge includes sensing resistive gauges for recording electrical	Tested on juvenile porcine eyes	While using this contact lens made application of any anesthetic drugs needless, while the vision remained	As the utilized materials (PC and PI) are nontransparent, it could have a negative impact on patient's field of view over	[7]

nector	Then, they were added in a Wheatstone bridge setup with two compensation resistive gauges and two sensing resistive gauges.	rounded arc-shaped around the center, to detect meticulously changes in IOP from corneal deformation.		signals.		clear.	long-term IOP measurements. Additionally, the ZIF connector is unpleasant to be carried by a patient.	
**Contact lens sensor**								
Flexible conducting all-organic BL films Electrical connections Pt-wires Copper wires	A thin film was cut from the central hole of a hard contact lens with doughnut-shaped by using polymerizable BF-2 glue, and attached to the whole rims of the CLs. Two annealed Pt-wires were utilized for the electrical connection and glued with graphite paste to the sensing layer of the BL film and were attached to two twisted copper wires, afterwards.	In this device, IOP variations were transferred via deformation of the soft contact lens to a sensor with Pt-based design.	IOP monitoring	Piezo-resist mechanism	Tested on an enucleated pig eye.	The device allows incorporating polymeric matrices of a wide-ranging mechanical property and some organic conductors while keeping their distinctive electromechanical details. In comparison to a standard silicone band, which was employed by conventional eye surgery, this BL film has higher biocompatibility.	The system is not able to use battery power, because of the decreased accessible area on the CLs.	[8]
**Soft chipless contact lens sensor**								
Soft silicone Embedded LC resonator Integrat-	The inductive coil was etched in a standard method. Then, a sacrificial layer of SU-8 photo-resist was spin-coated on a clean glass	Changes in the corneal curvature alter the embedded coils inductance. To detect wirelessly the fluctuations in the	IOP monitoring	A resonance circuit embedded inside the lens	A model eye made of silicone rubber.	The resonance frequency of the device changes with IOP	If it was thinner (now the thickness is <150 μm), it could represent	[9]

ed capacitor Inductive oil	substrate in order to release the inductive coil. Subsequently, a 500 nm copper seed layer was sputtered on the SU-8. A seed layer was spin-coated by a 30 μm resin and was patterned via lithography process on the contact lens. Finally, an ultrasonic bath was used to release the inductive coil.	coil inductance as changes in the LC resonance via an external reader, the inductive coil and a capacitor were connected to form a resonator. Thorough creating a relationship between LC resonance and IOP changes, the IOP variations could be calculated dynamically.		was designed to track the change of the corneal curvature.		alterations and with the lens curvature.	improved oxygen permeability and extended wear ability.	
**Implantable wireless pressure sensor**								
Hypodermic needle Micromachined capacitive pressure sensor Flexible polyimide coil	By using polyimide (Kapton tape) as insulator and copper as conductor, the coils were developed by standard flex cable technology. DRIE was used to etch device layer and to produce a capacitive gap to feed through holes. Before insulating the bulk silicon via a thin oxide layer from electrical connections, a thick layer of intermediated oxide was wet-etched on the contact lens using BOE. Also, MEMS methodology was used to develop the capacitive pressure sensors.	An external coil can wirelessly detect resonant frequency of the transponder by employing the phase-dip method, and meanwhile by using impedance analyzer, the external coil phase can be monitored. Then, the recording of a frequency sweep is performed, and the natural frequency of the transponder is indicated by the location of phase-minimum. To evaluate the resonant frequencies, an Agilent impedance analyzer was used with the phase dip method.	IOP monitoring	A micromachined capacitive pressure sensor connected to the needle back-end allows the pressure sensor to be in direct hydraulic contact with intraocular space.	Implanted *in-vivo* in rabbits for one-month	Implanting via the pars plana originates from the exclusive design of this structure that provides a direct hydraulic contact of pressure sensor with intraocular space, which delivers a real-time IOP evaluation.	The device needs surgery to be implanted inside the eyes.	[10]
**Flexible-coiled wireless IOP sensor**								
Flexible/foldable parylene disk substrate Pressure access hole of the dia-	Deep silicon etching and thermal oxidation multi-layer parylene micromachining were used to monolithically microfabricate the sensor. In order to describe the device release limitations, DRIE was conducted	Passive telemetric sensing can equip the contact lens with straightforward IOP measurement through using an embedded transensor that records environmental pressure changes	IOP monitoring	A pressure-sensitive parallel-plate variable capacitor.	*In-vivo* testing performed by a rabbit eye model and *ex-vivo* testing conducte	The sensor succeeds in an acceptable sensing distance that is appropriate for glasses reader	This device needs surgery to be implanted within the eyes.	[11]

phragm chamber Spiral inductor Metalline overpass Pressure-sensitive variable capacitor	on the backside of the wafer. Two thick titanium/gold layers were patterned by metal etching method. After conducting another DRIE followed by photoresist stripping with acetone, the final version of the device includes a backside recess for a larger volume of pressure capacity.	within the eye, and then IOP evaluation could be performed directly via an external wireless reader.			d by an enucleated porcine eye.	realization while suitable for minimally invasive implantation.		
**Implantable contact lens with CMOS pressure sensor**								
RF transponder Sensor signal processing unit Digital control unit	A CMOS method with a surface micromachining extension was used to develop the single-chip implant for the capacitive pressure sensors.	Transferring data and receiving power wirelessly was performed by the transponder, thus making battery-less process possible.	IOP monitoring	Micromechanical pressure detection	The proposed contact lens has not been tested.	Temperature measurement and wireless remote is provided by the system using RF transmission, which makes battery-less process possible.	The method is invasive as it requires implanting the contact lens within the eye.	[12]
**Implantable telemetric endosystem**								
Telemetry chip Read-out chip Pressure sensor Coil Capacitor	An eight-mask MOS-type procedure was performed to develop a capacitive absolute pressure sensor. Inside the microcavity of well is filled with reference pressure that works below 100 Pa and performed via low-pressure chemical vapor deposition sealing with low-temperature oxide. The fabricated chip was integrated into an intraocular lens.	For the output signal of sensor, the telemetry chip delivered a data reduction scheme via two 12-bit counters and the passive data modulation functionality. The technical idea of ITES originates from the inductive telemetric transmission of energy to an implanted device and data transferring by skin to an external measuring system and data logging unit by an approach of passive absorption	IOP monitoring	The measurement was achieved via sealing the microcavity below the pressure-sensitive membrane under low-pressure conditions.	The device is proposed to be subject to intensive clinical tests during cataract surgery.	There is no relation between battery lifespan and implant lifespan, and also there is no risk of pollution because of the battery presence.	The transmission method of this device is based on the short operating distance of a few millimeters that comes from the near-field effect of magnetic induction.	[13]

		modulation.						
**Wireless ocular telemetry sensor**								
Silicone contact lens<br>Embed-ded MEMS<br>Thin mi-crofabri-cated platinum titanium strain gauge	The OTS was an integrated MEMS silicone contact lens that was microfabricated with a thin platinum titanium strain gauge.	Fluctuations by corneal curvature generate IOP changes that were recorded by OTS. A mounted antenna surrounding the eye received and transmitted the data to a collector.	IOP monitori ng	Piezo-resist mechani sm	Tested on 13 patients for IOP monitorin g over 24-h.	The OTS represents a nice capability and an acceptable safety level for 24-h evaluation of IOP in patients.	An antenna is placed around the eye inside a soft patch, while the other part named recorder is located around the neck and the waist, which may pose inconvenienc e for the patient.	[14]
**Multifunctional smart soft contact lens device**								
PDMS<br>PDMS cure agent<br>Meth-acrylic ester<br>11-Mecaptou ndecanoic acid<br>8-Mercapto-1-Octanol<br>EDC<br>NHS<br>EA<br>PPA<br>PBS buff-er<br>IL-12p<br>FSS	For AAO sensor fabrication, through an E-beam evaporator the highly purified aluminum plotted onto the cover glass slide. A chrome steel ball was located on a preheated hotplate in order to fabricate the contact lens. The liquid PDMS was poured, and flowed down along the surface of the ball. 3 min later, PDMS was cut via pressure against the ball by a tube with a circular rim.	A layer of a transparent bio-compatible nano-porous thin film was integrated within the contact lens. The device is used as pressure sensor for monitoring the IOP. The biomarker detection sensor with coupled Abs and Ags caused a peak shift in optical signals. The nano-pores in the thin film served as nanocontainers for collecting drug and to facilitate the diffusion for extended drug release, a thin layer of porous silicone covered them.	IOP monitori ng, extende d *in-situ* drug delivery, and glaucom a biomark er IL-12p70 detectio n	In this device, 3 separate function al units/sen sors facilitate its operatio n. The pressure sensor was responsi ble for IOP measure ment. The optical sensor was used to detect the glaucom a biomark er. And the drug	Tested on a cadaver cornea of a pig.	The three main parts of the contact lens are fabricated from the same AAO thin film and optically transparent; therefore, considerabl y it leads to a simple fabrication process and reduces the cost.	The biomarker amount in the eye is limited, and it's impossible to detect glaucoma accurately solely relying on biomarker measurement s.	[15]

				was released via porous silicon barriers.			
**First-in-human continuous IOP and OPA contact lens**							
Micro-processor Antenna Pressure sensor	Medical-grade silicone elastomers were used to mold the PMCL with oxygen plasma to confirm the surface hydrophilicity. The MEMS pressure sensor was located in center, a telemetry microprocessor, and circular antennas were implanted in the contact lens.	The contact lens contains a medical-grade silicone elastomer that used to mold the PMCL to guarantee hydrophilicity. This device provides the IOP evaluations. The PMCL contact lens was positioned on the eye, and via a periorbital adhesive patch antenna sent wireless signals to a recorder. Finally, recordings were transferred to a computer for readout and visualization.	IOP and OPA monitoring	An individually calibrated MEMS sensor performed the measurements. The proprietary geometry of the lens that keeps it in position and relaxed the cornea, and facilitated the IOP measuring at 1Hz.	Tested on 8 human subjects for 24-h IOP and OPA measurements.	The lens is suitable for overnight wearing due to its hydrophobicity and can perform constant IOP evaluation in an ambulatory setting for 24-h period.	A simultaneous readout by PMCL and tonometry (GAT/DCT) is not possible as no reliable tonometry evaluation could be conducted with a contact lens placed on an eye.	[16]
**Stimulus-responsive contact lens**							
Polymer-izable surfactant Silmer A008-UP Aqueous phase liquid Nonaqueous phase liquid PNIPAM	The contact lens was fabricated with concentric patterns using photolithography. A moiré pattern was created on a metal platform using the normal lithography technique. Subsequently, the developed patterns were charged with ink,	The moiré effect was applied to monitor and measure IOP alterations. Through this method, a virtual image was used as a reference and was superimposed onto the generated micro pattern on the contact lens surface. A skew calibration and alignment of	IOP monitoring	A number of concentric circles on the patterns detect alterations of the eyeball diameter.	An animal test with white New Zealand rabbits was reported.	Drug elution was initiated by body temperature.	Any movement of eye or mispositioning of the contact lens could interpret shifted moiré. As long as the lens has adequate	[17]

	and using the stamping method the pattern was transferred to the actual device.	concentric images allowed the moiré image to be assessed HOG and Fourier transformation in the context of the applied pressure. The results were analyzed with MATLAB.					mechanical solidity and oxygen penetrability, it remains functional. An obvious decrease in IOP after using contact lens made this strategy highly promising for acute glaucoma treatment.	
**Non-invasive IOP monitoring contact lens**								
PDMS Dyed bio-compatible avocado oil Reference markers	A casting process was used to develop the contact lens. Surface treatments performed on two pieces of PDMS and then attached together to reach a thickness of 250 μm for contact lens. Dyed avocado oil that is a biocompatible material employed as the indicator fluid. Next, it was injected into a microchannel of 100 μm diameter that one side of it was left open to the atmosphere and the other side was sealed with PDMS.	Fluctuations in corneal curvature come from variations of IOP that results in deformation of an integrated microchannel and represents on the indicator fluid. In order to calibrate displacement measurements, some known reference markers of specific length was used. Finally, analyzing captured images revealed measurement of changes in IOP.	IOP monitoring	The changes in the level of indicator fluid were imaged and analyzed.	Tested on fresh enucleated porcine eyes.	The contact lens provided a reliable response with great linearity to IOP alteration. It met the requirements of anatomically different eyes while recording both increase and decrease in IOP throughout cyclical fluctuations.	To improve absolute and relative IOP recording, the contact lens structure requires a new design. Also, the device couldn't calculate IOP changes over the night and through sleep, besides it has to refine the fabrication polymers to increase durability for human eyes.	[18]
**Doughnut-shaped contact lens**								
RLC resonator circuit Constant on-chip capacitor Inductive antenna	Parylene-C was deposited, followed by the deposition of a patterned metal layer, then another layer of parylene-C, and again one more layer of metal layer was deposited. Subsequently, the third parylene-C layer was	Using soft PDMS for contact lens provides continuing normal vision of user and conformal assignment of the sensor over the curved cornea.	IOP monitoring	Changes in the corneal curvature results in bending of interior channels	*Ex-vivo* test on the canine eye was performed.	Having reliable parts, simple design, low price, and being highly responsive are some of the	The device has no enough durability and in return there is no appropriate response of the corneal and scleral	[19]

	deposited and the masking metal was created through photolithography patterning. After wet etching of fourth metal layer, the devices were located inside a contact lens of a doughnut shape via a PDMS molding method.			of PDMS lens, while these channel changes in the diameter, transfers the glycerol either backward or forward, therefore calculating IOP.		beneficial features of this device.	tissue to IOP variations, which represents a significant difference in *ex-vivo* and *in-vivo* experiments.	
**Strain sensing embedded contact lens**								
Closed microfluidic network<br>Gas reservoir<br>Liquid reservoir<br>Sensing channel	The PDMS device was fabricated by soft lithography techniques. The thin NOA65 films with the required features were made and bonded together to make sensors. For the fabrication of this device, specific fabrication methods were developed to build extremely thin (~ 100 um) microfluidic devices. The gas permeability of polyurethane used in devices is 6–8 orders of magnitude lower than metals used in wearable electronics.	The sensing channel established liquid-gas equilibrium. This would fluidically change as a response to the radius of curvature variations on a cornea or as a response to mechanical stretching and release of the cornea.	IOP monitoring	The liquid-gas equilibrium pressure interface and equilibrium were used for measuring the IOP.	Tested on enucleated porcine eyes.	It will also help in the regulation of synaptic plasticity of neurons as one of the critical factors for the progress of synaptic plasticity.	The stiffness, the comfort, and the lens-cornea mechanic interactions need to be optimized.	[20]
**Wireless smart contact lens for IOP evaluation**								
Antenna<br>Passive gauges<br>Telemetry microprocessor<br>Active gauges	The thin platinum-titanium IOP sensor embedded in the contact lens. The gauges were sandwiched between two polyimide layers. By using electrodeposition, the gold loop antenna was	The sensor has two sensing-resistive gauges to double the sensitivity of recording and is integrated within the lens to record the changes of corneal curvature. The portable glasses sent	IOP monitoring	An integrated sensor keeps track of cornea deformation that is produce	Tested on enucleated pig eyes.	It provides a minimally invasive procedure of constant monitoring of IOP for 24-h evaluation.	The complete package of contact lens comes with a pair of glasses or patch for measuring IOP during night that is	[21]

	added and then the microprocessor was attached by a flip-chip method. They were interconnected to the antenna and sensor strain gauges via conductive epoxy glue. Several steps of cast molding method formed the whole device.	energy to the lens and in return microprocessor sends back an output signal based on the IOP variations. Finally, the data were recorded and used for further evaluations.		d by IOP variations.			not comfortable for users to wear.	
**Power-less colorimetric smart contact lens**								
Microfluidic channel Parylene membrane Photonic crystal membrane	Casting method by employing a PDMS mold was used for fabrication of contact lens. A SU-8 mold that was prepared by photolithography utilized for replication of PDMS pattern of microfluidic channel. A hole was vertically created on the channel for the sensing part. Then the lens was heated at 100 ◦C and deposition of parylene was performed all over the parts except PC membrane, afterwards.	The pressure caused by corneal curvature transferred to parylene-coated ring-shaped fluidic channel within the contact part of the lens. This pressure induced fluid flow in the channel toward a small hole and moved the sensing part made of flexible PC membrane. Ultimately, deformation of PC membrane result in color changes that is easily detectable.	IOP monitoring	Sensing part detects the morphology fluctuations in the eyeball according to changes in IOP.	*In-vitro* study on silicone eye model and *ex-vivo* study on porcine eyeball	No need for battery power and an expensive optical spectrometer (a smart phone camera is enough)	Limit of detection measured via smartphone camera is not relatively high enough.	[22]
**Ultra-sensitive wearable contact lens sensor**								
Liquid reservoir Sensing channel Air reservoir	Standard photolithography method was utilized to develop the multilayer silicon molds. Also, standard soft lithography method was used to create duplicates of above-mentioned molds from PDMS. Overall, PDMS contact lens molds were produced on steel spherical balls that have a direct relationship with the radius of curvature of the porcine cornea.	The air reservoir, liquid reservoir, and sensing channel are interconnected. The axial release decreases the microfluidic liquid reservoir volume and vice versa, therefore, results in a vacuum effect. The vacuum push air-liquid interface to the liquid reservoir position. The difference of air-liquid interface position between each step could define the sensitivity	IOP monitoring	The working principle of the strain sensor is based on volume expansion of the microfluidic channel network.	Tested *ex-vivo* on an enucleated porcine eye	Using volumetric amplification could translate small strain changes into a large fluidic volume expansion that is easily detectable.	The liquid reservoir network is produced with high channel stiffness and it could decrease sensitivity of the sensor.	[23]

		as a function of 1% strain.						
**IOP monitoring contact lens with islet transplantation**								
Antenna Load coil Strain sensor Inductive coil	In the fabrication process, a SU8 epoxy layer was patterned as the rigid part, and integrated inside a silicone elastomeric layer. Then, a sacrificial layer was sputtered on a Si wafer as the soft contact lens material. Next, it was transferred onto the elastomeric layer for the creation of strain sensor. Meanwhile, both Si membranes were attached electrically with the load coil. Consequently, SU8 epoxy layer rigid patterns were coated to cover only the antenna area. Finally, the whole layers were molded into the shape of a contact lens.	An embedded strain sensor within the contact lens recognized minute fluctuations in intraocular pressure via focusing on the selective area of the contact lens. Then, the recorded IOP transferred by using an antenna to an outer device.	IOP monitori ng	An integrate d rigid ring pattern ensures IOP changes are received by strain sensor and monitore d.	*In-vivo* tested on STZ-induced diabetic Lewis rat.	The results of the contact lens represented a high correlation with the values of rebound tonometer.	After 4 weeks of Intraocular islet transplantati on, elevated IOP values returned to normal expectations.	[24]
**IOP continuously monitoring contact lens**								
Pressure sensor Tran-sponder compo-nents Signal condition-ing units Internal telemetric compo-nents RF tran-sponder	A pressure sensor was micromachined and integrated with signal conditioning units. A digital-to-analog converter in addition to an internal telemetric part for wireless communication was embedded on the chip monolithically. The transmission coil and chip are positioned on non-optical part of contact lens.	A capacitive absolute pressure sensor was placed in the central part of the microsystem via surface micromachining method. The electronics parts were monolithically integrated and convert the analogue signals to pulse-width-modulated signals, which in result could reduce pick-up noise and calculate IOP changes.	IOP monitori ng	Flexible material s provide d the folding properti es for the lens.	*In-vivo* experime nts were performe d	Both versions of this contact lens showed the same precision as widely accepted gold standard for the determinati on of the IOP	According to the influence of the silicone coating, a small offset was observed.	[25]
**Non-invasive highly sensitive graphene sensors for IOP measurement**								
Graphene layer	PDMS was spin-coated on silicon wafer and via	IOP changes come from the radial	IOP monitori	Piezo-resistive	Tested on a silicone	Representin g high	There are some factors	[26]

Wheat-stone bridge PDMS Parylene Strain gauge Compen-sating resistors	chemical vapor deposition the parylene was dropped on the PDMS film. Next, by UV light photolithography the photoresist was spin-coated and patterned on the parylene film. Finally, the IOP sensor was achieved vial placing the device into a metal mold and keeping it for 20 min on a 160 °C hot plate.	contraction or expansion of the cornea, and then two strain gauges on the radial direction and two compensating resistors on the edge of the sensor could detect it.	ng	eyeball with diameter of around 24 mm.	sensitivity and flexibility. Additionally, no cost-effective fabrication process.	that can affect contact lens function including blinking, corneal thickness, curvature, and biomechanics. Also, average transparency of 85% could be improved.	

Antibodies (Abs), Antigens (Ags), Bicontinuous microemulsion nanoporous contact lenses (BME-CLs), Bi-layer (BL), Anodic aluminum oxide (AAO), Buffered oxide etchant (BOE), Carbon nanotubes (CNTs), Complementary metal oxide semiconductor (CMOS), Contact lens sensor (CLS), Deep-reactive-ion-etching (DRIE), Ethanolamine (EA), Fluorescein sodium salt (FSS), Graphene-silver nanowire (AgNW), Histogram of gradient (HOG), Implantable Telemetric Endosystem (ITES), Inductive coil with a capacitor (LC), Intraocular pressure (IOP), Metal nanowires (mNWs), Micro-electro-mechanical system (MEMS), Microtransfer molding (μTM), *N*-(3-Dimethylaminopropyl)-*N*′-ethylcarbodiimide hydrochloride (EDC), *N*-Hydroxysuccinimide (NHS), Norland Optical Adhesive 65 (NOA65), Ocular pulse amplitude (OPA), Ocular telemetry sensor (OTS), Phosphoric acid (PPA), Polyimide (PI), Platinum (Pt), Platinum-titanium (PtITi), Polydimethylsiloxane (PDMS), Polyethylene terephthalate (PET), Polyethylene terephthalate (PETE), Polycarbonate (PC), Poly(*N*-isopropylacrylamide) (PNIPAM), Pressure-measuring contact lens (PMCL), Reactive ion etching (RIE), Streptozotocin (STZ), Titanium (Ti), Ultraviolet (UV), Zero Insertion Force (ZIF).

Table S2. Contact lenses for Glucose measurement and monitoring. The table summarizes all the existing platforms including their key parts, the process of fabrication, the operation mechanism, the working principle and development stage, as well as the advantages and disadvantages.

Main components	Fabrication strategy	Mechanism of operation	Application	Development stage	Advantages	Disadvantages or limitations	Refs.
			**Contact lens biosensor**				
Insulator mem-brane PDMS mem-brane Pt elec-trode Flexible Ag/AgCl electrode	A silicon wafer was deposited by a PDMS membrane through spin-coating for 30 min at 80 ◦C. The sputtering technique was used to add a Pt film over a PDMS layer by a titanium stencil that was developed via a laser microfabrication process.	Enzymatic reactions induce hydrogen peroxide concentrations and in result the biosensor could measure glucose concentration. Subsequently, after decreasing the standard glucose solution was to the initial value, the output current instantly increased.	Glucose monitoring	Pre-clinical stage	The biosensor effectively completed both measurements in the temporal calculation of tear glucose and in constant evaluation of tear dynamics in preclinical animal tests.	Tear fluid bio-components like ascorbic acid and other proteins can affect the biosensor results.	[27]
			**CMOS glucose sensor**				
Loop an-	The wafer was cut from	A PCB was used to	Glucose	Not	The device is	On the one	[28]

tenna Wireless sensor interface chip Glucose sensor on a polymer substrate	PET films and cleaned, and was subsequently coated with a positive photoresist that was deposited on the surface by spin coating. Routine photolithography steps were followed. Acetone used to lift off the evaporated Cr, Ni, and Au. DI water mixed with Au etches to etch the seed layer. By using nitrogen gas, the wafer was dried and then via using a CO laser cutter, the individual contact lenses were cut out. Then the contact lens was aligned with the chip over it.	integrate the chip and sensor on it and in order to describe performance and functionality. Three electrodes were applied for the electrochemical sensor, one as a working electrode, another one for counter electrode, and the last one for a reference electrode.	monitoring	mentioned	powered wirelessly and the sensitivity detection range is 0.18 μA for glucose.	hand, in receiving wireless power at low frequencies, it is restricted by the antenna efficiency, and on the other hand the path loss dominates the wireless power at high frequencies.	
**Contact lenses based on photonic structure sensor**							
AA BIS 3-APBA DMSO DEAP β-D-(+) glucose PBS	The PS master was used as a stamp and by using drop-casting technique, the monomer solution was deposited on it. UV polymerization conducted on the monomer solution, and then the stamp replica was peeled off from the master PS. A photonic micro-pattern that was treated with phenylboronic acid was created on a Glucose-selective hydrogel layer.	When binding with glucose, the photonic structure began to swell, modulating the periodicity constant.	Glucose monitoring	Tested on an artificial eye.	The fabrication method is simple and straightforward, while the measurement does not need sophisticated or expensive coupled devices. The sensor contact lens is reliable and biocompatible.	The device is not suggested for patients who deal with IOP-induced ocular diseases.	[29]
**A smart contact lens**							
Hybrid substrate Rectifier LED Glucose sensor A transparent, stretchable conductor	To develop a transparent and stretchable antenna for transmitting power wirelessly, the electrospun fibers of AgNFs were deposited on the platforms at the same time as the rectifier circuit was built. A combination of photolithography and a wet etching technique was used for used to	An electric signal was transferred to the contact lens by antenna to further activate the glucose sensor and the LED pixel. A real-time sensing data was transmitted upon detecting glucose concentrations. If the glucose level of tear proceeds above the	Glucose monitoring	Tested on New Zealand rabbits	The contact lens enables the real-time and wireless functioning.	When the LED pixel turns off, it was unclear whether it is due to the overly concentrated glucose content or damage to the circuit.	[30]

	remove a sacrificial layer that allowed the wireless to display.	threshold, then results in turned off pixel.					
**IOP-glucose contact lens**							
Graphene CNTs mNWs AgNW Resistance circuitIn-ductance circuit Capaci-tance cir-cuit	The plastic substrate such as PETE and PDMS were bent on cylindrical supports with various radii of curvature. The contact lens was equipped with a stretchable and transparent electrode. Using photolithography technique, Graphene was applied onto the AgNW electrodes to form hybrid electrodes and channel.	Between the main factors involved in the RLC circuit resistance was correlated to molecular binding while inductance and capacitance change with structural alterations of the device, enabling IOP detection.	Glucose and IOP monitorin g	Tested *in-vitro* on a bovine eyeball for IOP measure ment and *in-vivo* on a male New Zealand white rabbit for Glucose monitorin g.	Specific binding of the target analyte to graphene was made possible.	Precise diagnosis of glucose may require further development of the sensor.	[2]
**Contact lens for diagnosis and therapy of diabetes**							
f-DDS Wireless power ASIC chip Remote commu-nication system HEMA EGDMA	On-demand f-DDS was made by the laser lift-off technique. Plasma-enhanced CVD was applied to develop hydrogenated amorphous silicon exfoliation and SiO2 layers. Anode and cathode electrodes were treated by e-beam evaporation and lithography. The XeCl excimer laser was emitted on the backside of the glass substrate to detach the drug reservoir on the PET film from the glass substrate.	The reader coil was connected to a commercial power amplifier and wirelessly transferred the electrical power to the contact lens for real-time glucose sensing within the tear. The wireless transmission was performed by using a custom-made ASK receiver module, an AVR, and a PC.	Glucose monitorin g and on-demand controlled drug delivery	Tested on white New Zealand rabbits.	The device provides electrically controlled on-demand drug delivery through wireless power and remote communications targeting diabetic diagnosis and therapy.	The contact lens did not observe the time-lagged increase of glucose concentrations between the blood and the tear.	[31]
**Ultrathin MoS2 transistor integrated contact lens**							
TMDC semicon-ductors Elastic intercon-nect elec-trodes Polyimide pas-sivation layers	In the fabrication process, a gold-mediated exfoliation and assembly strategy was employed to pattern mono-layered MoS2 flakes. A layer of PI was patterned on the MoS2 transistors for mechanical reinforcement. And by using aserpentine gold	First, GOD was immobilized on the MoS2 surface. In the oxidation reaction of glucose with GOD, hydrogen ions and electrons were produced. The free electrons resulted in an increase of device current. Then a pure	A glucose sensor, a temperatu re sensor for detecting corneal disease, and a photodete ctor for	*In-vitro* cytotoxicit y tests were conducte d	The serpentine mesh sensor could directly mount onto the lens and without interfering vision or blinking could deliver high sensitivity and mechanically robust feature.	As an external force, curvature-induced stress could affect on the radial and longitudinal direction of contact lens and result in local buckling.	[32]

PDMS lens substrate MoS2 transistors	and PI mesh structure, the elastic parts were created. Finally, in order to avoid any blocking vision, the sensor was positioned around the outer ring of the cornea.	phosphate-buffered saline solution increased the concentration to the same level of glucose concentration typically found in human tear fluid, while the current glucose concentration is compared with diabetic tear fluid glucose level.	receiving optical information				
**Paper microfluidic contact lens**							
HEMA GOD Phosphomolybdic Commercial contact lens Tris HCl Tris base Sodium fluorescein	Colorimetric sensors were fabricated first of all and then placed on paper, and created a paper microfluidic sensor. Next, it was integrated within a commercial laser contact lens. Overall, the microfluidic chip was coated by a poly-HEMA contact lens that was chemically bonded in laboratory.	Paper matrices contained chemical sensors were integrated within contact lens structures. In order to perform the detection, based on tear analytes changes in concentration, the chromogenic sensors produced a deviation in the primary reflected wavelength that is a kind of visible spectrum and could be detected by user's cellphone camera.	Detection of glucose, proteins, hydrogen ions, nitrites and L-ascorbic acid	*In-vitro* measurements were conducted for colorimetric assays	Simultaneous evaluation directly from eye tears for biomarkers/proxies including glucose, proteins, ascorbic acid, nitrite ions and pH.	The response of an animal body to foreign device and immunogenicity was not assessed in *in-vivo* and *in-vitro* samples.	[33]
**Gelated colloidal crystal contact lens**							
3-D polystyrene CCA PVA RGP contact lens	The monomers of PS were prepared with a diameter of less than 200 nm. The RPG lenses by ultrasonic cleaning method were hydrophilized with deionized water for 12 h and dried. Then, by using vertical sedimentation technique PS monomers were self-assembled on RPG	Sensing system works based on selectively cause the visible light diffraction. Changing glucose concentration resulted in diffraction of light wavelength as a clear indicator. It works for glucose concentration range	Glucose monitoring	*In-vitro* tests were performed	The sensor was able to specifically find and attach to glucose in the presence of other analytes that resulted in a weak response of ~10 nm.	The capability of sensor lens in response to glucose concentrations was limited to shift only between 567 nm and 468 nm.	[34]

	contact lens. Finally, at a continuous 60 °C temperature, PS particles altered into colloidal crystal on the contact lens surface.	between 0 to 50 mM and the visible light turn into reddish yellow, green, and blue.					
**Holographic glucose sensor for non-Invasive contact lens**							
Nelfilcon A ® Darocur 1173 Holo-graphic wafer	A holographic wafer with 7 μm thick and 3 mm diameter was attached with Nefilcon A contact lens meanwhile the polymerization procedure was performing. To design a holographic glucose sensor, the boronic acid ligands, combined into a polyacrylamide matrix. After the illumination of hologram, the fringes reflect a narrow band of color.	Glucose binding to the boronic acid caused hydrogel swelling which resulted in color change and glucose concentration detection, which finally is diagnosed by the photofluorometer near to the eye.	Glucose measure ment and refractive error correction	Pre-clinical stage	Receiving multiple readings holographic signal simply, without pain, and non-invasively.	There could be some false results, as the boronic acid method of binding is not as precise as the other methods like fluorescent technique.	[35]
**Fluorescent glucose sensor contact lens**							
TRITC-Con A FITC-dextran Nelfilcon A ® Darocur 1173	TRITC-Con A and FITC-dextran were added to the procedure of contact lens polymerization. Then, Nelfilcon A® went under cross-link polymerization by Darocur 1173. in order to eliminate any dissolved oxygen, nitrogen gas was purged afterward. An ultraviolet light stream was performed for edges refinement. Contact lens fabrication was finished after 6 h, and to remove any unpolymerized monomers the molds were then soaked in MilliQ® water overnight.	On the binding site on TRITC-Con A, when the glucose level increased, glucose took the FITC-dextran place and at the same time fluorescence rised proportional to glucose level.	Glucose monitorin g	*In-vitro* tests were performe d	The examination on corneas represented no symptoms of abrasion, cloudiness, or damage.	The results represented a delay of glucose measurement in time between blood glucose and contact lens fluorescence.	[36]
**Boronic acid doped contact lens**							
BAF PVA Millipore water	A range of boronic acid with fluorophores sensors were synthesized and tested.	In the presence of glucose, based on the perturbation of the charge transfer	Glucose monitorin g	Pre-clinical stage	No reports of probe leaching and a shelf-life of more than	The contact lens responded for only 20 % of available tear	[37]

	These sensors were able to function in low pH and methanol-like polarity of an off-the-shelf plastic contact lens. Then, a proto-type glucose sensing contact lens was developed according to boronic acid containing fluorophores integration with a commercial contact lens.	nature of the excited states, the spectral changes could be detected.			several months.	glucose in 50–500 μM range.	

2,2-diethoxyacetophenone (DEAP), 3-(acrylamido)phenylboronic acid (3-APBA), Acrylamide (AA), Alf Vegard Risc (AVR), Amplitude shift keying (ASK), Application-specific integrated circuit (ASIC), Boronic acid containing fluorophores (BAFs), Crystalline colloidal array (CCA), Carbon nanotubes (CNTs), Chemical vapor deposition (CVD), Dimethylsulfoxide (DMSO), Effective isotropically radiated power (EIRP), Flexible drug delivery system (f-DDS), Fluorescein isothiocyanate–dextran (FITC-dextran), Glucose oxidase (GOD), Graphene-silver nanowire (AgNW), Metal nanowires (mNWs), Molybdenum disulfide (MoS2), *N,N*-methylenebis(acrylamide) (BIS), Phosphate-buffered saline (PBS), Photonic structure (PS), Polydimethylsiloxane (PDMS), Polyethylene terephthalate (PET), Poly(vinyl alcohol) (PVA), Reactive ion etching (RIE), Rigid gas permeable (RGP), Tetramethylrhodamine isothiocyanate–concanavalin A (TRITC-Con A), Transition metal dichalcogenide (TMDC).

Table S3. Contact lenses for colorblindness applications. The table summarizes all the existing platforms including their key parts, the process of fabrication, the operation mechanism, the working principle and development stage, as well as the advantages and disadvantages.

Main components	Fabrication strategy	Mechanism of operation	Application	Development stage	Advantages	Disadvantages or limitations	Refs.
**Gold nanoparticle contact lens**							
HEMA<br>EGDMA<br>Photoinitiator<br>Gold nanoparticles	First, gold nanoparticles were stabilized in phosphate buffer solution. In order to make sure that the nanoparticles were evenly distributed within the polymer, the polymer was sonicated for 30 min, which also helped to separate additional parts that formed as a result of the centrifugation. Subsequently, the solution was inserted into the	Gold nanoparticles filtered the variety of optical wavelengths that CVD patients were hardly able to distinguish between particular colors.	CVD treatment	*In-vitro* study was conducted	The gold nanoparticles contact lens represented superior water retention and wettability features in comparison with available commercial CVD wearables.	Oxygen permeability test must have performed as HEMA hydrogels could absorb oxygen via water molecules.	[38]

	contact lens mold and treated with UV light for 5-10 min. Finally, to remove all of the residues; the contact lens was washed twice with DI water.						
**Silver nanoparticle contact lens**							
EGDMA HEMA Photoinitia-tor Silver nano-particles	The contact lenses loaded with silver nanoparticles were fabricated by mixing HEMA, EGDMA, and photo-initiator. In order to ensure complete homogenization, the solution was added to the glass cuvette for sonication over 30 min. Then the desired number of nanoparticles were mixed and It was polymerized via UV lamp for 5-10 min followed by adding the whole solution into a contact lens mold. Finally, it was washed by DI water.	According to spherical silver nanoparticles size and dielectric properties, they attract visible light of 390-490 nm ranges and reduce color distinction for blue-yellow CVD patients.	Treatment of blue-yellow CVD patients	*In-vitro* study was performed	Experiments represented a good effectiveness of color filtering and biocompatible properties of contact lens.	Some nanoparticle clusters were created and some nanoparticles were unevenly dispersed that could result in changing transmission spectrum of the nanoparticles.	[39]
**Color blindness contact lens**							
Rhodamine dye DMSO pHEMA	A fluorescent rhodamine dye was used for the wavelength-filtering feature of contact lens. To apply the dye on contact lens, the drop and dip method was used to cast a drop of the dye directly on the lens surface and dip the	The dye could filter out light wavelength between 545 and 575 nm to correct color vision blindness.	CVD treatment	*In-vitro* study was conducted	The contact lens indicated no toxicity and 99% cell viability of human corneal epithelial cells after 72 h.	The main drawback was that the dye diffused in the PBS solution easily, which is a sort of leakage.	[40]

	contact lens into the dye solutions for 1 min.							

Color vision deficiency (CVD), Deionized water (DI water), Dimethyl sulfoxide (DMSO), Ethylene glycol dimethacrylate (EGDMA), Hydroxyethyl methacrylate (HEMA), poly(2-hydroxyethyl methacrylate) (pHEMA).

Table S4. Contact lenses for drug delivery applications. The table summarizes all the existing platforms including their key parts, the process of fabrication, the operation mechanism, the working principle and development stage, as well as the advantages and disadvantages.

Main components	Fabrication strategy	Application	Mechanism of operation	Type of drug	Development stage	Advantages	Disadvantages or limitations	Refs.
			**Contact lens for DES**					
HEMA EGDMA MAA Darocur® HA	Free radical polymerization was used to develop an implant/ring was fabricated by utilizing a pre-monomer mixture thorough the modified cast molding technique. Then glass plate mold was located in a UV transilluminator, and irradiating at 365-370 nm polymerized the sheet. Then the removed sheet of HA-laden was molded by borer to develop the ring/implant.	Delivering continuous ocular HA within the contact lenses for DES treatment.	HA was dispersed from the hydrogel implant slowly via the lens matrix when the contact lens is positioned on the eye, following by moving from the post lens and pre-lens tear film.	HA	Tested on white New Zealand rabbits	The device reduces systemic exposure, improves the bioavailability of drug, and increase patient adherence for improved clinical results.	The complete drug leach-out was not achieved, and some amount of HA was locked up inside the implant permanently or could be leach-out but wasn't recognized via applied colorimetric technique.	[41]
			**Drug-eluting contact lens for DES**					
HA (TBA)–OH Dowex resin Ethylenediamine Cholesteryl chlo-	EGDMA was used as a cross-linker to develop a commercial contact lens by photopolymerization of HEMA. The final mixed solution (cyclosporine/C–HA) was used	A drug-eluting contact lens to treat DES by containing C–HA micelles.	The nanoparticles were filled with the hydrophobic drugs and were diffused by the structure of micellar design in the contact lens hydrogel and subsequently diffused via the	Cyclosporine	Tested on white New Zealand rabbits	The continuously delivered drug through contact lens shows better efficiency in comparison to eye drops.	The transparency decreased (ca. 90%) compared to normal contact lenses (ca. 95%) because of the C–HA micelles that could affect visual field.	[42]

roformate HEMA EGDMA Cyclo-sporine Insulin EGF Hydro-cortisone Chlera-toxin	for injection to a mold of contact lens and for 15 min it was cured at 340 nm under a nitrogen environment.		contact lens hydrogel.					
**Contact lens for fungal keratitis**								
HTCC Silver nanopar-ticles GO Vor Antifun-gal drug	The hydrogel contact lens was developed inside a lens mold to which the solution of HTCC was injected to cast the hydrogel. The through electrostatic force between GO and HTCC facilitated the cross-linking of the hydrogel hence strong mechanical integrity of the contact lens.	This antifungal structure represents a considerable potential for fungal keratitis treatment in an effective and rapid way.	Contact lens provides antimicrobial functions due to having HTCC and silver nanoparticles; while for the drug carrier, GO plays the role and meanwhile employs the anionic group of HTCC for building electrostatic crosslinking with the cationic group.	Vor	Tested by left eye of mice that has fungal keratitis	This sustained drug delivery system has desirable softness and flexibility, and exhibited excellent *in-vitro* and *in-vivo* cytocompatibility and great antimicrobial functionality.	After 24 h, the drug release rate becomes slow, which is not efficient.	[43]
**Bimatoprost imprinted silicone contact lens**								
Bimato-prost HEMA EGDMA Irgacure® DMA Meth-acrylic acid Siloxane	Monomer solution was used for casting the silicone contact lenses. The mixture of solution was spiked in the male and female mold. The assembly was transferred to a UV transilluminator and cured at 370–380 nm for 15 min. In order to eliminate the	The goal of contact lens was not only increasing the loading level of bimatoprost from the soaking solution, but also maintaining the drug delivery to treat Glaucoma.	The drug was mixed with the monomers, and followed by drug-loaded contact lens development. After diffusing ocular drug during the monomer sterilization and extraction, the tailored active sites of drug pockets were left behind that demonstrated a great potential of	Bimatoprost	Tested on white New Zealand rabbits	The device not only improves the level of bimatoprost loading from drug-soaking solution, but also sustains drug delivery from lens matrix.	The great ability of contact lens drug uptake has a direct adverse effect on the ion and oxygen permeability.	[44]

	monomers that didn’t react with contact lens, it was immersed for 30 min in boiling water.		loading ocular drugs from the drug-soaking solution.					
**TM loaded microemulsion laden silicone contact lens**								
TM HEMA EGDMA Irgacure® DMA Siloxane	Fabrication process of silicone contact lenses were conducted by solution of monomers added into the female and the male molds, which were attached cautiously. Then it was moved to a UV transilluminator and cured at 350–380 nm for 15 min.	The contact lens was aimed at investigating the result of swelling and TM uptake from the solution of TM-microemulsion soaking, TM delivery mechanism from contact lens and optical transmission to manage Glaucoma.	Microemulsion showed a great capacity of drug uptake, and contact lens design toward lens matrix in comparison to the soaking solution could increase the TM-microemulsion uptake. The continuous TM delivery to the eye originates from the tight surfactant interface at oil-water.	TM	Tested on white New Zealand rabbits	The device improved the TM uptake and delivery mechanism without altering main properties of the contact lens.	The presence of TM-microemulsion might alter the contact lens permeability of oxygen and ions.	[45]
**Multifunctional smart soft contact lens device**								
PDMS PDMS cure agent Meth-acrylic ester 11-Mecapto undeca-noic acid 8-Mercap-to-1-Octanol EDC NHS EA PPA PBS buff-er IL-12p FSS	For AAO sensor fabrication, an E-beam evaporator employed to deposit highly purified aluminum on the cover of glass slide. For contact lens fabrication, a chrome steel ball was located on a preheated hotplate and the liquid PDMS was poured on the steel ball and flows down slowly over the ball surface. After 3 min, PDMS was cut via pressing a	The device was aimed at IOP measurement, extended *in-situ* release of ocular drugs, and IL- 12p70 glaucoma biomarker detection.	The nanocontainers provided a place for storing and releasing drugs, which a thin layer of porous silicone covered them as a permeation impediment for continuous drug delivery.	TM	Pre-clinical stage	Three main parts of the contact lens are fabricated from the same optically transparent and AAO thin film that considerably decrease the price and simplify the process of creation.	There was no constant drug release ratio, as it was increased with rise in IOP level.	[15]

	tube with a circular rim against the ball. Finally, the typical thickness of the contact lens turned out to be ~120 μm.							
**Bicontinuous microemulsion contact lens**								
GMA HEMA 3-[tris(trimethylsiloxy) sily] propyl methac-rylate DMA 2-hydroxy-2-methylpropiophe-none EGDMA AIPH	During PEO-RMA-40 contact lens development procedure, the nonaqueous phase was treated with ultrasound and then vortexed, followed by the addition of water and AIPH. Next, the mixture inserted to a mold of contact lens for polymerization thermally for 1 h at 60 °C in an oven. For the Silmer-based BMCLs fabrication, the aqueous phase was vortexed and then nonaqueous phase added to the solution mixture. Finally, DMA was added. UV polymerization conducted in a PDMS casting mold exposed to UV light.	Loading and release of TM	Temperature-sensitive drug-delivery both in nanogels-laden and in isolated nanogels contact lenses recommended that swelling and shrinking behavior were reversible. Every procedure could repeat after frequent cooling and heating. The task for temperature-responsive gatekeeper is provided by the outer shell of nanogels for the ocular drugs that electrostatically stuck within the matrix of *p*(NIPAM).	TM	Pre-clinical stage	Thermosensitive pNIPAM nanogelsladen contact lenses represented thermal activated continued drug delivery at 35 °C. Additionally, the optical transmission and permeability of oxygen remained the same after loading with DLNG.	The *in-vivo* results might be different due to the presence of tear film proteins	[46]
**Drug-implants-laden silicone contact lens**								
HEMA Irgacure® EGDMA NVP	Female molds were used to place the drugs in a radial	The device was aimed at upholding the release	The layers that are loaded with bimatoprost, TM, and HA were	Bimatoprost, TM, HA	Tested on white New Zealand rabbits	The device was preservative-free, allowed	Drug could be trapped permanently inside the	[47]

DMA Siloxane TM Bimato-prost HA	distance of 3 mm from center. Also, additional part of monomer mixture was poured into female mold and attached to male mold carefully. Then the assembly was placed in the UV transilluminator and by UV-B light cured at 360-370 nm for 30 min. Finally, the contact lenses were detached and kept at 25 °C.	rate minus from high initial burst delivery.	integrated within the exterior edge of the contact lens allowing the central part to remain clear. The drug burst was avoided by entrapping the drugs within the implant's matrix. The gradual release of HA, which acts as a physical lubricant, offered comfort to the patient's eyes over an extended period of time.			user-friendliness, and prevented initial drug burst. It is an excellent candidate for glaucoma treatment.	contact lens network and it could avoid complete delivery.	
**PVP coated sparfloxacin loaded ring contact lens**								
Sp PVP-K30 HEMA EGDMA Darocur® Siloxane	The contact lens was fabricated using the soaking method by mixing various monomers including Darocur®, EGDMA, HEMA, and siloxane. The ring was integrated within the edge of the silicone contact lens. The loading of Sp took place via soaking, direct loading, and ring casting techniques. The surface of the device was coated with PVP using the short surface curing technique.	The contact lens was aimed to cure conjunctivitis by co-delivering PVP and Sp for a continuous time.	The Sp was released into the eye upon systematic swelling of PVP while the optical transparency remained intact.	Sp	Tested on white New Zealand rabbits	Using PVP for covering outer surface of the contact lens avoids from pink eye syndrome and also delivers comfort to the patient eye.	The release rate of soaked contact lens was very high. The stability of the loaded drug might be questionable since the contact lens showed SP leaching.	[47]
**PVP loaded olopatadine HCl contact lens**								

OL<br>Ethyl cellulose<br>PVP-K30<br>HEMA<br>Darocur®<br>EGDMA<br>DMA<br>Siloxane	The olopatadine HCl-loaded doughnut-shaped ring was implanted within the periphery of the lens using the modified casting technique. The loading of OL was performed via soaking, direct loading, and doughnut casting methods. A curing technique allowed deposition of PVP on the surface of the contact lens.	The device was aimed at releasing OL and PVP without affecting the optical and swelling properties of contact lenses to treat allergic conjunctivitis.	The sustained release was being achieved by entrapping the drug in the matrix of ethylcellulose microparticles. The PVP will provide comfort to the patient eye, avoiding pink eye syndrome. Therefore, the new doughnut shape contact lens that is PVP-loaded olopatadine-ethyl cellulose microparticles laden could provide a better option to treat allergic conjunctivitis.	OL	Tested on white New Zealand rabbits	The PVP provides comfort to the patient eye, avoiding pink eye syndrome.	The stability of the loaded OL over time might be questionable since signs of leaching from the contact lens was observed	[48]
**Prednisolone nanoparticle contact lens**								
Predniso-lone<br>PLGA<br>PVA<br>DCM<br>HEMA<br>EGDMA<br>MAA	The three-polymeric mixture including HEMA (80%), MAA (19%) and EGDMA (1%) were stirred for contact lens fabrication. Above-mentioned values were injected into casting molds and were thermally polymerized at 80 °C. Then, the optimized nanoparticles were added into the contact lens matrix.	The system was developed for topical ocular administratio n of prednisolone.	Delivering the drug into the tear fluid was performed by drug diffusion through the polymeric matrix of the nano particles.	Predniso lone	*In-vitro* study was conducted	The contact lenses remained transparent after incorporation with nanoparticles and displayed a good drug release over 24 h.	An initial slow ratio of drug release happened due to the drug capture in nanoparticles.	[49]
**Self-reporting colorimetric contact lens**								
Silica na-noparti-	The molds were prepared and	Loading and release of TM	The binding nanocavities sites	TM	Drug release	The contact lens could	PH level changes could	[50]

cles HEMA MAA TM EGDMA HMPP	imprinted precursor was added. Then the UV irradiation polymerization was performed on it. Next, the SiO_2 layer and imprinted molecule templates were removed that lead into the colored contact lens with a thickness of around 0.1 mm in the center.		were created from guest-molecule patterns in polymer matrix, and have a great binding affinity for molecular recognition. The process of binding or unbinding of TM molecules generated the response of hydrogel matrix. These responses could be converted to operational optical signals through the photonic crystal structure.		was tested in an artificial tear fluid.	self-report the TM delivery by colorimetric assay	affect and decrease TM binding into the imprinted sites and in result to release ratio.	
**Magnetic micropump contact lens**								
PDMS MNPC Magnetic micro pump Ostemer layer	By utilizing standard photolithography technique, the PDMS master mold was fabricated. Using Ostemer 322 and the PDMS master mold the main body of the micropump was casted. O_2 plasma treatment was performed for total assembly of the magnetic micropump. Finally, the whole device was punched via biopsy punches.	The proposed micropump could provide an on-demand drug delivery.	By using a short exposure of the magnetic field, the membrane assembly deflection was induced. At the same time the micro check valve began to be opened by magnetic field. Next, the drug diffused to the area inside the micro check valve. The valve was entirely opened after 0.06 s, and drug began to leave the micropump and after 0.76 s, the release was completed, and the check valve was closed.	Not mentioned specifically but could be any appropriate ocular drug.	Pre-clinical stage	The contact lens could be precisely controlled for drug delivery in nanoliter scale by opening and closing the micro check valve magnetically.	Further studies on model eyes or animals should be conducted for bio-compatibly safety.	[51]
**Soft contact lens for sustained delivery of Timolol**								
HEMA EGDMA	Hydrogels were prepared by	Release administratio	By immersing the dry hydrogels in	TM	*In-vitro* study was	Loading capacity of	During the release process,	[52]

MAA AIBN TM	mixing HEMA, EGDMA, MAA with or without TM. Then the solution was injected in the molds and was cured at 50 °C in an oven. Boiling process removed unreacted reagents to produce a dry hydrogel with smooth, clear and poreless surface.	n of TM on soft contact lenses	the release medium, TM was released through changes in the dimensions of the gel matrix.		conducted	TM was increased while maintaining suitable release characteristics.	if water absorbed faster than TM in the hydrogel contact lens, then it could diffuse through the swollen gel instead of TM.	
**Antifungal contact lens**								
PLGA Irgacure Econa-zole HEMA EGDMA Glucose Histidine Leucine	PLGA was dissolved in ethyl acetate and vortexed by adding econazole. Ethyl acetate was eliminated via evaporation and the provided film was coated with pHEMA via UV radiation. Subsequently, it was moved into a lens mold to be polymerized with UV over 60 min.	The contact lens aimed for extended release of econazole as an antifungal drug.	Drug was released in phosphate-buffered saline medium by continuous shaking.	Econazo le	*In-vitro* study was conducted	The contact lens was functional over the 3 weeks.	In case the contact lens is removed from eye, the components will be degraded as the lens will continue to release drug.	[53]
**Soft contact lens with molecular imprinted design**								
DEAA MAA EGDMA Darocure TM	MAA, EGDMA, TM and Darocure were mixed, and the solution was added into a polypropylene contact lens mold. Then, the free radical polymerization was performed with UV	Continuous release of TM in precorneal area of the eye	TM-loaded contact lens diffuse TM via the matrix of contact lens and slowly enter the TM to post-lens tear film, which finally result in trapping between the cornea and the contact lens.	TM	*In-vivo* tests performed on male Nippon albino rabbits	The molecular imprinted contact lens provides more drug loading capacity and was able to release constantly TM into eye.	The contact lenses based on DEAA materials have not a great loading capacity.	[54]

	irradiation at room temperature over 20 min.							
**Temperature triggered contact lens**								
EGDMA<br>PGT<br>HEMA<br>PBS<br>MMA	Free radical polymerization was used to mix HEMA, and TM loaded nanoparticles in order to synthesize the particle-laden *p*-HEMA gels. Next, photoinitiator was added and transferred into a lens mold. Then, it was irradiated with UV light over 40 min.	TM delivery for ophthalmic diseases	The drug release happened based on hydrolysis of ester bonds between TM and contact lens matrix.	TM	*In-vitro* study was conducted	Drug release period was extended from 1-2 h to 2-4 weeks.	There is the possibility of drug release in the packaging solution, if the contact lens is not carefully rinsed before using on the eyes.	[55]
**Diamond embedded contact lens for therapeutic purposes**								
Nanodi-amond-nanogels<br>HEMA<br>MAA<br>Darocure<br>EGDMA	First, the nanodiamonds were coated with polyethyleneimine for cross-linking with a polysaccharide to create a nanodiamond-nanogel that is loaded with TM. Then, they were integrated into a HEMA matrix and poured to contact lens mold.	Delivery of glaucoma drugs, such as TM	Getting in touch with tear fluid can cut the chitosan and release the TM.	TM	*In-vitro* study was conducted	The contact lens represents a new era for contact lenses capable of enzyme-triggered drug release.	Some burst release was seen after 48 h of lysozyme treatment.	[56]
**Nanoparticle loaded contact lens for drug delivery**								
PCL<br>HEMA<br>PEG-DA	The nanoparticles were provided via surfactant-free miniemulsion polymerization. Then, HEMA	Delivering loteprednol etabonate eye drop as a hydrophobic drug	First the drug has diffused from nanoparticles into the HEMA hydrogel and then delivered into the tear fluid.	Loteprednol Etabonate	*In-vitro* study was conducted	Extended drug delivery of loteprednol etabonate up to 12 days.	Incorporating of nanoparticles into contact lens caused oxygen permeability reduction and lens modulus increase.	[57]

	based hydrogel was generated at room temperature with free radical photopolymerization. The resulted solution was poured into the contact lens mold and irradiated by UV light over 30 min.							
				**Natamycin embedded contact lens**				
HEMA DMAA EGDMA DMSO Natamycin Photoinitiator	EGDMA and photoinitiator were mixed and stirred over 5 min. Then, the resulting mixture transferred into an aluminum contact lens mold to be cured over 30 min via UV light. Finally, they were hydrated in 100 mL DI water overnight and were cut in circle disks before use.	Loading and release of antifungal drug natamycin.	Drug-nanoparticles release from contact lens started from delivery of nanoparticles from hydrogel network to hydrogel aqueous phase. Then they are delivered into tear fluid.	Natamycin	*In-vitro* study was conducted	The contact lens represented an extended drug delivery up to 12 h for specific drug release directly to cornea of the eye.	A burst release was observed in the first 1 h of drug delivery.	[58]

2–20-azobis [2-(2-imidazolin-2-yl) propane] dihydrochloride (AIPH), Antibodies (Abs), Antigens (Ags), Bicontinuous microemulsion contact lens (BMCL), Bimatoprost-soaked contact lenses (BT-SM), Anodic aluminum oxide (AAO), Chitosan (CS), Contact lens (CL), Cyclosporine-loaded cholesterol-HA (C–HA), Dry eye syndrome (DES), Dichloromethane (DCM), Dimethyl acrylamide (DMA), Epithelial growth factor (EGF), Ethanolamine (EA), Ethylene glycol dimethacrylate (EGDMA), Fluorescein sodium salt (FSS), Glycidyl methacrylate (GMA), Graphene oxide (GO), Hyaluronic acid (HA), Hydroxylethylmethacrylate (HEMA), 2-hydroxy-2-methyl-1-phenyl-1-propanone (HMPP), Interleukin 12 (IL-12p), Intraocular pressure (IOP), Magnetic nanoparticle-PDMS composite (MNPC), Methacrylic acid (MAA), *N*-(3-Dimethylaminopropyl)-N′-ethylcarbodiimide hydrochloride (EDC), *N*-Hydroxysuccinimide (NHS), *N*,*N*-dimethylacrylamide (DMAA), *N*-isopropylacrylamide (NIPAM), *N*-vinyl pyrrolidone (NVP), *N*,*N*-diethylacrylamide (DEAA), Olopatadine HCl (OL), Phosphate-buffered saline (PBS), Phosphoric acid (PPA), Poly ε-caprolactone (PCL), Poly ethylene glycol diacrylate (PEG-DA), Polydimethylsiloxane (PDMS), Polyvinyl alcohol (PVA), Polyvinylpyrrolidone (PVP), Poly DL-lactic-co-glycolic acid (PLGA), PGT (propoxylated glyceryl triacylate), Quaternized chitosan (HTCC), Silicon dioxide (SiO2), Simulated tear fluid (STF), Sparfloxacin (Sp), Tetrabutylammonium (TBA), Timolol maleate (TM), Ultraviolet (UV), Voriconazole (Vor).

Table S5: Electrospun fiber-based contact lenses for drug delivery applications. The table summarizes all the existing platforms including their key parts, the process of fabrication, the operation

mechanism, the working principle and development stage, as well as the advantages and disadvantages.

Type of fiber	Main components	Electrospinning strategy	Application	Development stage	Advantages	Disadvantages or limitations	Refs.
**Electrospun TM-loaded polymeric contact lens**							
Polymeric nanofiber	PVP PNIPAM Ethanol BAC EDTA Borneol PureVision® Balafilcon A silicone hydrogel contact lenses Brij® 78	The polymer solution including 5% w/v of PVP-PNIPAM with ratio of 1:1 ratio was dissolved in ethanol and later two various concentrations of TM including 5% w/w and 15% w/w were drawn into the syringe for fiber disposition. The solution infusion was done via silicone tubing in a flow rate of 8 to 15 µL/min by a single stainless needle of 1.6 mm inner diameter.	A contact lens of TM drug delivery for glaucoma treatment	Pre-clinical stage	This design addresses shortcomings related to poor drug availability when compared with other contact lenses.	The permeation enhancers may act as an extra barrier to drug diffusion.	[59]
**Improved contact lens utilizing polymer electrospinning (patent)**							
Fibrils of natural human corneal stromal collagen	Polymer fibril scaffold "mat" polymer Solute suitable solvent	Collagen was dissolved in a suitable solvent and under hydrostatic pressure inserted to a needle with conductive properties. 4,000-12,000 volts of DC potential was chosen according to the distance and desired fiber diameter and kept between the needle and the target that is placed away from the needle. The distance should be enough appropriate to preclude production of a coronal discharge or arc.	Improving permeable contact lens that mimics the native corneal structure.	No drug has been tested. The platform has the potential to be used for drug delivery applications.	The contact lens improves comfort of user, lipid deposition, and delays protein. It also could be used for drug delivery.	The electrospinning setup could undergo further improvements with respect to the technical aspects and expenses involved. Specifically, to neutralize the charge on the polymer jet's surface and minimize the Coulomb repulsion, a source of free ions can be used.	[60]
**Electrospun nanofibers for ocular drug delivery**							
Mix of pullulan and low acetylated	Pullulan Low acetylated gellun gum Kelcogel® GG-LA	Gellan gum was blended in different ratios with 15% pullulan (or higher). After adjustment of the gellan gum content, the blended solutions of 0.225% gellan gum and 15% pullulan resulted in the formation of fibers. The	A new solid *in-situ* gel-based delivery system for the treatment	No drug has been tested. The platform has the potential	The fluorescein residence time was extended when compared	A fine-tuning of pullulan concentration is required to avoid the polymer	[61]

gellun gum Kelcogel® GG-LA	FS STF	electrospun 20% pullulan 0.225% gellan gum fibers were shaped into lens with a height of 2.54 mm ± 0.38 mm. The curvature forming process reduced the lens diameter by 1 mm to 1.4 cm.	of topical ocular diseases	to be used for drug delivery applications.	with conventional eye drops.	stream from breaking into droplets of forming beaded fibers.	
**Chitosan-based nanocoatings ocular contact lenses**							
PVP and PNIPAM	PVP PNIPAM Chitosan ethanol TM Acetone Sodium hydroxide Rhodamine B PE PureVision® Balafilcon A silicone hydrogel contact lenses	A 2.5% w/w PVP and 2.5% w/w PNIPAM was dissolved in ethanol to prepare a 5% w/w polymeric solution as the base solution for making a series of 20 mL formulations. Each solution includes different chitosan concentrations and with respect to polymer concentration it contains a 15% w/w of TM. Via the EHDA system, a 5 mL BD Plastipak™ Luer syringe was placed over an Elite syringe infusion pump containing the polymer-drug solution. As the working distance, 12 cm under the needle, a collector plate was located which is recognized by exploratory experiments. At an ambient temperature of 23 °C, the flow rate of 10 mL/min at 17.8 kV was used.	Delivery of TM over extended periods.	*In-vitro* drug release	The contact lens provides alternative dosage management, specifically aiming for elder population with glaucoma, among which is poor compliance with conventional TM eye solutions.	Drugs could have trapped inside the polymer crystalline network (chitosan), therefore incomplete release.	[62]
**Electrically atomized formulations of ocular lens coatings**							
PVP and PNIPAM	PVP TM Acetone Sodium hydroxide Rhodamine B PureVision® Balafilcon A silicone hydrogel contact lenses	An on-demand and controlled fiber depositing were performed using single needle EHD method.	On-demand nano-structured delivery of TM	*In-vitro* studies on probe release and drug release were performed.	The contact lens is a good candidate for decreasing the dosing regimen, which results in lower reduce systemic drug absorption.	The composite materials have shown signs of degradation at a lower temperature in comparison to polymer-drug platforms.	[63]
**Extended-release drug–delivery contact lenses (patent)**							
Mat of polymer fibers	HEMA/MA EGDMA cross-linker AIBN initiator Triton X-100	No detailed electrospinning method is disclosed for this proprietary platform.	Simultaneous lipid permeability and impermeability while offering a high oxygen permeability	NS	The device exhibits the diffusive and natural optical properties of corneal tissue.	The integration of the electrospun nanofiber mat with contact lens may result in expanded	[64]

	DI water Polyvinyl alcohol		for drug delivery			inconsistently.	
**Functionalized contact lens coatings engineered**							
PVP and PNIPAM	PVP PNIPAM Ethanol TM BAC EDTA Brij® 78 Borneol	A syringe infusion pump was used to diffuse the solution that was injected to the 5 mL syringe. The electrospinning setup permitted to control flow rate of liquid through the pump. A conductive stainless-steel needle was connected to a high-power voltage supply to diffuse the solution that was coming from the silicone tubing. The resulting coatings were deposited directly on dehydrated silicone contact lens of PureVision Balafilcon A.	The device was aimed at releasing TM from electrically atomized coatings.	Pre-clinical stage	The device allows an increased diffusion of drug via cornea and therefore improves bioavailability of ocular drugs by using EHDA.	The insoluble EDTA can behave as a diffusional barrier hampering the TM relocation throughout the polymeric matrix towards the corneal membrane.	[65]

Azoisobutyronitrile (AIBN), Benzalkonium chloride (BAC), Borneol (PE), Deionized water (DI water), Electrohydrodynamic (EHD), Electrohydrodynamic atomisation (EHDA), Ethylene glycol dimethacrylate (EGDMA), Ethylenediaminetetraacetic acid (EDTA), Fluorescein sodium (FS), Hydroxyethyl methacrylate (HEMA), Methacrylamide (MA), Poly (*N*-isopropylacrylamide) (PNIPAM), Polyvinylpyrrolidone (PVP), Simulated tear fluid (STF), Timolol maleate (TM).

References

1. Pang, Y.; Li, Y.; Wang, X.; Qi, C.; Yang, Y.; Ren, T.-L. A contact lens promising for non-invasive continuous intraocular pressure monitoring. *RSC Adv.* **2019**, *9*, 5076–5082.
2. Kim, M.; Lee, M.-S.; Kim, K.; Ji, S.; Kim, Y.-T.; Park, J.; Na, K.; Bae, K.-H.; Kim, H.K. Wearable smart sensor systems integrated on soft contact lenses for wireless ocular diagnostics. *Nat. Commun.* **2017**, *8*, 14997
3. P. Auvray, L. Rousseau, G. Lissorgues, F. Soulier, O. Potin, S. Bernard, F. Dieuleveult, E. Scorsone, P. Bergonzo, L. Chicaud, A passive pressure sensor for continuously measuring the intraocular pressure in glaucomatous patients, IRBM 33(2) (2012) 117-122.
4. I.E. Araci, B. Su, S.R. Quake, Y. Mandel, An implantable microfluidic device for self-monitoring of intraocular pressure, Nature medicine 20(9) (2014) 1074.
5. M. Leonardi, P. Leuenberger, D. Bertrand, A. Bertsch, P. Renaud, A soft contact lens with a MEMS strain gage embedded for intraocular pressure monitoring, TRANSDUCERS'03. 12th International Conference on Solid-State Sensors, Actuators and Microsystems. Digest of Technical Papers (Cat. No. 03TH8664), IEEE, 2003, pp. 1043-1046.
6. G.-Z. Chen, I.-S. Chan, D.C. Lam, Capacitive contact lens sensor for continuous non-invasive intraocular pressure monitoring, Sensors and Actuators A: Physical 203 (2013) 112-118.
7. M. Leonardi, P. Leuenberger, D. Bertrand, A. Bertsch, P. Renaud, First steps toward noninvasive intraocular pressure monitoring with a sensing contact lens, Investigative ophthalmology & visual science 45(9) (2004) 3113-3117.
8. V. Laukhin, I. Sánchez, A. Moya, E. Laukhina, R. Martin, F. Ussa, C. Rovira, A. Guimera, R. Villa, J. Aguiló, Non-invasive intraocular pressure monitoring with a contact lens engineered with a nanostructured polymeric sensing film, Sensors and Actuators A: Physical 170(1-2) (2011) 36-43.
9. G.-Z. Chen, I.-S. Chan, L.K. Leung, D.C. Lam, Soft wearable contact lens sensor for continuous intraocular pressure monitoring, Medical engineering & physics 36(9) (2014) 1134-1139.
10. G. Chitnis, T. Maleki, B.C. Samuels, L.B. Cantor, B. Ziaie, Minimally Invasive Implantable Wireless Pressure Sensor for Continuous IOP Monitoring, Investigative Ophthalmology & Visual Science 52(14) (2011) 666-666.
11. P.-J. Chen, S. Saati, R. Varma, M.S. Humayun, Y.-C. Tai, Implantable Flexible-Coiled Wireless Intraocular Pressure Sensor, (2009).
12. K. Stangel, S. Kolnsberg, D. Hammerschmidt, B. Hosticka, H. Trieu, W. Mokwa, A programmable intraocular CMOS pressure sensor system implant, IEEE Journal of Solid-State Circuits 36(7) (2001) 1094-1100.
13. T. Eggers, J. Draeger, K. Hille, C. Marschner, P. Stegmaier, J. Binder, R. Laur, Wireless intra-ocular pressure monitoring system integrated into an artificial lens, 1st Annual International IEEE-EMBS Special Topic Conference on Microtechnologies in Medicine and Biology. Proceedings (Cat. No. 00EX451), IEEE, 2000, pp. 466-469.
14. K. Mansouri, T. Shaarawy, Continuous intraocular pressure monitoring with a wireless ocular telemetry sensor: Initial clinical experience in patients with open angle glaucoma, British Journal of Ophthalmology 95(5) (2011) 627-629.

15. C. Song, G. Ben-Shlomo, L. Que, A Multifunctional Smart Soft Contact Lens Device Enabled by Nanopore Thin Film for Glaucoma Diagnostics and In Situ Drug Delivery, Journal of Microelectromechanical Systems 28(5) (2019) 810-816.
16. R. Wasilewicz, T. Varidel, S. Simon-Zoula, M. Schlund, S. Cerboni, K. Mansouri, First-in-human continuous 24-hour measurement of intraocular pressure and ocular pulsation using a novel contact lens sensor, British Journal of Ophthalmology (2020).
17. S.-H. Lee, K.-S. Shin, J.-W. Kim, J.-Y. Kang, J.-K. Kim, Stimulus-Responsive Contact Lens for IOP Measurement or Temperature-Triggered Drug Release, Translational Vision Science & Technology 9(4) (2020) 1-1.
18. A. Campigotto, S. Leahy, G. Zhao, R.J. Campbell, Y. Lai, Non-invasive Intraocular pressure monitoring with contact lens, British Journal of Ophthalmology (2019) bjophthalmol-2018-313714.
19. M.H.M. Kouhani, J. Wu, A. Tavakoli, A.J. Weber, W. Li, Wireless, passive strain sensor in a doughnut-shaped contact lens for continuous non-invasive self-monitoring of intraocular pressure, Lab on a Chip (2020).
20. I.E. Araci, S. Agaoglu, M. Baday, P. Diep, Closed Microfluidic Network for Strain Sensing Embedded in a Contact Lens to Monitor IntraOcular Pressure, Google Patents, 2019.
21. M. Leonardi, E.M. Pitchon, A. Bertsch, P. Renaud, A. Mermoud, Wireless contact lens sensor for intraocular pressure monitoring: Assessment on enucleated pig eyes, Acta ophthalmologica 87(4) (2009) 433-437.
22. B. Maeng, H.-k. Chang, J. Park, Photonic crystal-based smart contact lens for continuous intraocular pressure monitoring, Lab on a Chip 20(10) (2020) 1740-1750.
23. S. Agaoglu, P. Diep, M. Martini, K. Samudhyatha, M. Baday, I.E. Araci, Ultra-sensitive microfluidic wearable strain sensor for intraocular pressure monitoring, Lab on a Chip 18(22) (2018) 3471-3483.
24. J. Kim, J. Kim, M. Ku, E. Cha, S. Ju, W.Y. Park, K.H. Kim, D.W. Kim, P.-O. Berggren, J.-U. Park, Intraocular pressure monitoring following islet transplantation to the anterior chamber of the eye, Nano letters 20(3) (2019) 1517-1525.
25. U. Schnakenberg, P. Walter, G. Vom Bögel, C. Krüger, H. Lüdtke-Handjery, H. Richter, W. Specht, P. Ruokonen, W. Mokwa, Initial investigations on systems for measuring intraocular pressure, Sensors and Actuators A: Physical 85(1-3) (2000) 287-291.
26. J. Xu, T. Cui, T. Hirtz, Y. Qiao, X. Li, F. Zhong, X. Han, Y. Yang, S. Zhang, T.-L. Ren, Highly Transparent and Sensitive Graphene Sensors for Continuous and Non-invasive Intraocular Pressure Monitoring, ACS applied materials & interfaces 12(16) (2020) 18375-18384.
27. M.X. Chu, K. Miyajima, D. Takahashi, T. Arakawa, K. Sano, S.-i. Sawada, H. Kudo, Y. Iwasaki, K. Akiyoshi, M. Mochizuki, Soft contact lens biosensor for in situ monitoring of tear glucose as non-invasive blood sugar assessment, Talanta 83(3) (2011) 960-965.
28. Y.-T. Liao, H. Yao, A. Lingley, B. Parviz, B.P. Otis, A 3-$\mu\hbox {W} $ CMOS Glucose Sensor for Wireless Contact-Lens Tear Glucose Monitoring, IEEE Journal of Solid-State Circuits 47(1) (2011) 335-344.
29. M. Elsherif, M.U. Hassan, A.K. Yetisen, H. Butt, Wearable contact lens biosensors for continuous glucose monitoring using smartphones, ACS nano 12(6) (2018) 5452-5462.
30. J. Park, J. Kim, S.-Y. Kim, W.H. Cheong, J. Jang, Y.-G. Park, K. Na, Y.-T. Kim, J.H. Heo, C.Y. Lee, Soft, smart contact lenses with integrations of wireless circuits, glucose sensors, and displays, Science advances 4(1) (2018) eaap9841.
31. S.-K. Kim, J. Koo, G.-H. Lee, C. Jeon, J.W. Mok, B.H. Mun, K.J. Lee, E. Kamrani, C.-K. Joo, S. Shin, Wireless smart contact lens for diabetic diagnosis and therapy, Science Advances 6(17) (2020) eaba3252.
32. S. Guo, K. Wu, C. Li, H. Wang, Z. Sun, D. Xi, S. Zhang, W. Ding, M.E. Zaghloul, C. Wang, Integrated contact lens sensor system based on multifunctional ultrathin MoS2 transistors, Matter 4(3) (2021) 969-985.
33. R. Moreddu, M. Elsherif, H. Adams, D. Moschou, M.F. Cordeiro, J.S. Wolffsohn, D. Vigolo, H. Butt, J.M. Cooper, A.K. Yetisen, Integration of paper microfluidic sensors into contact lenses for tear fluid analysis, Lab on a Chip 20(21) (2020) 3970-3979.
34. J.-L. Ruan, C. Chen, J.-H. Shen, X.-L. Zhao, S.-H. Qian, Z.-G. Zhu, A gelated colloidal crystal attached lens for noninvasive continuous monitoring of tear glucose, Polymers 9(4) (2017) 125.
35. A. Domschke, W.F. March, S. Kabilan, C. Lowe, Initial clinical testing of a holographic non-invasive contact lens glucose sensor, Diabetes technology & therapeutics 8(1) (2006) 89-93.
36. W.F. March, A. Mueller, P. Herbrechtsmeier, Clinical trial of a noninvasive contact lens glucose sensor, Diabetes technology & therapeutics 6(6) (2004) 782-789.
37. R. Badugu, J.R. Lakowicz, C.D. Geddes, A glucose-sensing contact lens: A new approach to noninvasive continuous physiological glucose monitoring, Optical Fibers and Sensors for Medical Applications IV, International Society for Optics and Photonics, 2004, pp. 234-245.
38. A.E. Salih, M. Elsherif, F. Alam, A.K. Yetisen, H. Butt, Gold Nanocomposite Contact Lenses for Color Blindness Management, ACS nano 15(3) (2021) 4870-4880.
39. A.E. Salih, A. Shanti, M. Elsherif, F. Alam, S. Lee, K. Polychronopoulou, F. Almaskari, H. Al Safar, A.K. Yetisen, H. Butt, Silver Nanoparticle-Loaded Contact Lenses for Blue-Yellow Color Vision Deficiency, physica status solidi (a).
40. A.R. Badawy, M.U. Hassan, M. Elsherif, Z. Ahmed, A.K. Yetisen, H. Butt, Contact lenses for color blindness, Advanced healthcare materials 7(12) (2018) 1800152.
41. F.A. Maulvi, A.A. Shaikh, D.H. Lakdawala, A.R. Desai, M.M. Pandya, S.S. Singhania, R.J. Vaidya, K.M. Ranch, B.A. Vyas, D.O. Shah, Design and optimization of a novel implantation technology in contact lenses for the treatment of dry eye syndrome: In vitro and in vivo evaluation, Acta biomaterialia 53 (2017) 211-221.

42. J. Mun, J. won Mok, S. Jeong, S. Cho, C.-K. Joo, S.K. Hahn, Drug-eluting contact lens containing cyclosporine-loaded cholesterol-hyaluronate micelles for dry eye syndrome, RSC advances 9(29) (2019) 16578-16585.
43. J.-F. Huang, J. Zhong, G.-P. Chen, Z.-T. Lin, Y. Deng, Y.-L. Liu, P.-Y. Cao, B. Wang, Y. Wei, T. Wu, A hydrogel-based hybrid theranostic contact lens for fungal keratitis, ACS nano 10(7) (2016) 6464-6473.
44. F. Yan, Y. Liu, S. Han, Q. Zhao, N. Liu, Bimatoprost Imprinted Silicone Contact Lens to Treat Glaucoma, AAPS PharmSciTech 21(2) (2020) 63.
45. N. Wei, H. Dang, C. Huang, Y. Sheng, Timolol loaded microemulsion laden silicone contact lens to manage glaucoma: In vitro and in vivo studies, Journal of Dispersion Science and Technology (2020) 1-9.
46. S.H. Lee, H.J. Kim, D.H. Kim, W.S. Chang, T.P. Vales, J.W. Kim, K.H. Kim, J.K. Kim, Thermo-sensitive nanogel-laden bicontinuous microemulsion drug-eluting contact lenses, Journal of Biomedical Materials Research Part B: Applied Biomaterials 107(4) (2019) 1159-1169.
47. A.R. Desai, F.A. Maulvi, D.M. Desai, M.R. Shukla, K.M. Ranch, B.A. Vyas, S.A. Shah, S. Sandeman, D.O. Shah, Multiple drug delivery from the drug-implants-laden silicone contact lens: Addressing the issue of burst drug release, Materials Science and Engineering: C (2020) 110885.
48. Y. Xue, W. Zhang, Y. Lei, M. Dang, Novel Polyvinyl Pyrrolidone–Loaded Olopatadine HCl–Laden Doughnut Contact Lens to Treat Allergic Conjunctivitis, Journal of Pharmaceutical Sciences (2020).
49. A. ElShaer, S. Mustafa, M. Kasar, S. Thapa, B. Ghatora, R.G. Alany, Nanoparticle-laden contact lens for controlled ocular delivery of prednisolone: Formulation optimization using statistical experimental design, Pharmaceutics 8(2) (2016) 14.
50. J. Deng, S. Chen, J. Chen, H. Ding, D. Deng, Z. Xie, Self-reporting colorimetric analysis of drug release by molecular imprinted structural color contact lens, ACS applied materials & interfaces 10(40) (2018) 34611-34617.
51. C. Wang, J. Park, Magnetic micropump embedded in contact lens for on-demand drug delivery, Micro and Nano Systems Letters 8(1) (2020) 1-6.
52. C. Alvarez-Lorenzo, H. Hiratani, J.L. Gomez-Amoza, R. Martínez-Pacheco, C. Souto, A. Concheiro, Soft contact lenses capable of sustained delivery of timolol, Journal of pharmaceutical sciences 91(10) (2002) 2182-2192.
53. J.B. Ciolino, S.P. Hudson, A.N. Mobbs, T.R. Hoare, N.G. Iwata, G.R. Fink, D.S. Kohane, A prototype antifungal contact lens, Investigative ophthalmology & visual science 52(9) (2011) 6286-6291.
54. H. Hiratani, A. Fujiwara, Y. Tamiya, Y. Mizutani, C. Alvarez-Lorenzo, Ocular release of timolol from molecularly imprinted soft contact lenses, Biomaterials 26(11) (2005) 1293-1298.
55. H.J. Jung, A. Chauhan, Temperature sensitive contact lenses for triggered ophthalmic drug delivery, Biomaterials 33(7) (2012) 2289-2300.
56. H.-J. Kim, K. Zhang, L. Moore, D. Ho, Diamond nanogel-embedded contact lenses mediate lysozyme-dependent therapeutic release, ACS nano 8(3) (2014) 2998-3005.
57. F.H. Nasr, S. Khoee, M.M. Dehghan, S.S. Chaleshtori, A. Shafiee, Preparation and evaluation of contact lenses embedded with polycaprolactone-based nanoparticles for ocular drug delivery, Biomacromolecules 17(2) (2016) 485-495.
58. C.-M. Phan, L. Subbaraman, S. Liu, F. Gu, L. Jones, In vitro uptake and release of natamycin Dex-b-PLA nanoparticles from model contact lens materials, Journal of Biomaterials Science, Polymer Edition 25(1) (2014) 18-31.
59. P. Mehta, A.A. Al-Kinani, M.S. Arshad, M.-W. Chang, R.G. Alany, Z. Ahmad, Development and characterisation of electrospun timolol maleate-loaded polymeric contact lens coatings containing various permeation enhancers, International journal of pharmaceutics 532(1) (2017) 408-420.
60. R. Fuerst, J. Bango, J. Fenn, M. Dziekan, Fabrication of improved contact lens utilizing polymer electrospinning, Google Patents, 2009.
61. B. Göttel, J.M.d.S. e Silva, C.S. de Oliveira, F. Syrowatka, M. Fiorentzis, A. Viestenz, A. Viestenz, K. Mäder, Electrospun nanofibers–A promising solid in-situ gelling alternative for ocular drug delivery, European Journal of Pharmaceutics and Biopharmaceutics 146 (2020) 125-132.
62. P. Mehta, A.A. Al-Kinani, M.S. Arshad, N. Singh, S.M. van der Merwe, M.-W. Chang, R.G. Alany, Z. Ahmad, Engineering and development of chitosan-based Nanocoatings for Ocular Contact Lenses, Journal of pharmaceutical sciences 108(4) (2019) 1540-1551.
63. P. Mehta, A.A. Al-Kinani, R. Haj-Ahmad, M.S. Arshad, M.-W. Chang, R.G. Alany, Z. Ahmad, Electrically atomised formulations of timolol maleate for direct and on-demand ocular lens coatings, European Journal of Pharmaceutics and Biopharmaceutics 119 (2017) 170-184.
64. K. Davis, M. Reuter, A. Kammerich, A. Tangonan, K. Vedantham, A. Kelley, Extended release drug-delivery contact lenses and methods of making, Google Patents, 2018.
65. P. Mehta, A.A. Al-Kinani, O. Qutachi, M.S. Arshad, A. Alqahtani, M.-W. Chang, W.M. Amoaku, R.G. Alany, Z. Ahmad, Assessing the ex vivo permeation behaviour of functionalised contact lens coatings engineered using an electrohydrodynamic technique, Journal of Physics: Materials 2(1) (2018) 014002.
